# Vasculitis in Cystic Fibrosis

**DOI:** 10.3389/fped.2020.585275

**Published:** 2020-11-12

**Authors:** Francesca Sposito, Paul S. McNamara, Christian M. Hedrich

**Affiliations:** ^1^Department of Women's and Children's Health, Institute of Life Course and Medical Sciences, University of Liverpool, Liverpool, United Kingdom; ^2^Department of Paediatric Rheumatology, Alder Hey Children's National Health Service Foundation Trust Hospital, Liverpool, United Kingdom

**Keywords:** cystic fibrosis, vasculitis, immune complex, inflammation, pathophysiology, treatment, damage

## Abstract

Cystic fibrosis (CF) is an autosomal-recessive multi-organ disease characterized by airways obstruction, recurrent infections, and systemic inflammation. Vasculitis is a severe complication of CF that affects 2–3% of CF patients and is generally associated with poor prognosis. Various pathogenic mechanisms may be involved in the development of CF-related vasculitis. Bacterial colonization leads to persistent activation of neutrophilic granulocytes, inflammation and damage, contributing to the production of antineutrophil cytoplasmic autoantibodies (ANCAs). The presence of ANCA may on the other hand predispose to bacterial colonization and infection, likely entertaining a vicious circle amplifying inflammation and damage. As a result, in CF-associated vasculitis, ongoing inflammation, immune cell activation, the presence of pathogens, and the use of numerous medications may lead to immune complex formation and deposition, subsequently causing leukocytoclastic vasculitis. Published individual case reports and small case series suggest that patients with CF-associated vasculitis require immune modulating treatment, including non-steroidal anti-inflammatory drugs (NSAIDs), corticosteroids, hydroxychloroquine, and/or disease-modifying anti-rheumatic drugs (DMARDs). As immunosuppression increases the risk of infection and/or malignancy, which are both already increased in people with CF, possible alternative medications may involve the blockade of individual cytokine or inflammatory pathways, or the use of novel CFTR modulators. This review summarizes molecular alterations involved in CF-associated vasculitis, clinical presentation, and complications, as well as currently available and future treatment options.

## Introduction

Cystic fibrosis (CF) is the most common autosomal-recessive disease in White Caucasian populations, affecting 1 in every 2,500–3,500 new-borns (1). It is caused by mutations in the cystic fibrosis transmembrane regulator (*CFTR*) gene, located on the long arm of chromosome 7. This gene encodes for an ATP-binding chloride channel that is expressed on different cell types, including, but not limited to, airway epithelial cells (2). The CFTR ion channel contributes to and maintains the composition and the amount of liquid covering mucous layers throughout the body. More than 2,000 mutations have been identified that can affect *CFTR* mRNA and protein synthesis, its maturation, sub-cellular trafficking, and channel activity (3). Alterations in CFTR activity lead to defective chloride and bicarbonate secretion combined with increased sodium adsorption and mucus secretion. In the airway epithelium, this results in dehydration and acidification of the airway surface that causes impaired mucociliary clearance, recurrent infections and uncontrolled chronic inflammation leading to bronchiectasis, the main cause of morbidity and mortality in people with CF (pwCF) (4–6). However, CF pathology is not limited to the airways; CF is a multi-organ disease that also affects gastrointestinal, reproductive and endocrine functions amongst others (7).

**Figure 1:**
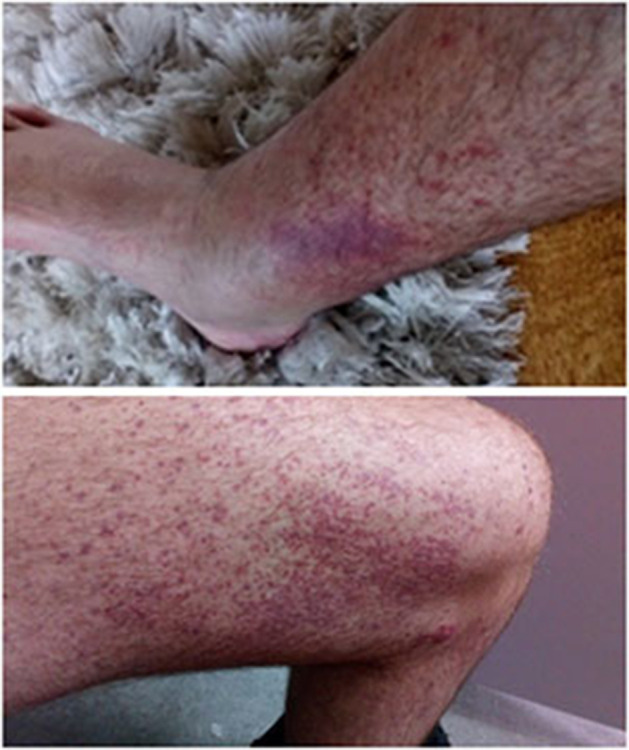
Palpable purpuric rash on lower limbs of a patient with CF-associated vasculitis.

Systemic vasculitis is a rare, but potentially severe complication of CF which can involve any organ system, but most commonly the skin (8). It involves venules, capillaries, arterioles and (sometimes) larger blood vessels (9). Several pathogenic mechanisms have been implicated in the induction of vasculitis in pwCF. Small vessel vasculitis in CF frequently involves the presence of antineutrophil cytoplasmic autoantibodies (ANCA) and the formation of immune complexes (ICs), whose deposition leads to leukocytoclastic vasculitis that is characterized by dense neutrophil infiltrates and complement C3 deposits within blood vessel walls in the papillary dermis (9–11) (Figure 1). Relatively few published reports in this area indicate that cutaneous and/or systemic vasculitis in pwCF is associated with poor prognosis with as many as 75–90% of pwCF and a diagnosis of vasculitis dying within 2 years (8, 12). Evidence based and/or targeted-directed individualized treatments do not exist.

The aim of this review is to summarize reports of vasculitis in CF, its molecular pathophysiology, and available and future treatment options.

## The Molecular Pathophysiology of Vasculitis in CF

### Immune Complexes in Inflammation and Tissue Damage

The pathophysiology of CF related vasculitis is not completely understood. It may be associated with bacterial colonization, deposition of immune complexes (ICs), hyper-gammaglobulinemia, and/or the effect of the numerous drugs that pwCF are administered (e.g., antibiotics, diuretics, non-steroidal anti-inflammatory drugs (NSAIDs), anticonvulsants, etc.).

Hyper-gammaglobulinemia and the presence of ICs in pwCF may be caused by systemic inflammation, even in the absence of self-directed autoimmune responses (13). *Per se*, IC formation is a physiological mechanism as it is the result of antibody production (IgG or IgM) and their binding to molecular targets, resulting in antigen neutralization. However, occasionally, as a result of increased ICs production or defective C3 clearance, ICs can deposit in vessels and tissues, where they activate Fcγ receptors (e.g., blood vessel walls, kidneys, and joints) and complement factors, resulting in immune cell recruitment and activation, inflammation and, finally, tissue damage. During this process, the localization of IC deposition determines symptoms and complications (14, 15). In addition, ICs, after complement cascade activation, induce the generation of complement effectors that can interact with neutrophils and stimulate a particular form of cell death called NETosis (16).

In CF, several factors may contribute to IC deposition: chronic bacterial and/or viral infection, the presence of autoantibodies, medication exposure, chronic inflammation, and tissue damage (17, 18).

Chronic bacterial infections trigger antibody production, including autoantibodies, thereby increasing ICs formation and overburdening ICs clearance by phagocytic cells (18). In this, ANCA antibodies, that can be found in CF patients and that will be discussed in detail below, may be of special interest, as they contribute to increased bacterial colonization and their presence can contribute to IC formation (19). Furthermore, medications may stimulate ICs production or interfere with ICs clearance, thus leading to ICs accumulation and deposition (20). In particular, antibiotics such as penicillins and cephalosporins, can cause ICs deposition in blood vessel walls leading to the development of leukocytoclastic vasculitis. This so-called type III hypersensitivity reaction is mediated by the deposition of drug-containing IC that fail to be removed after precipitation (21–23). Lastly, chronic inflammation results in cell and tissue damage that causes uncontrolled release of intracellular components to the extracellular space. This can result in autoantibody production, immune complex formation and deposition, and lastly vasculitis (15).

### The Role of Antineutrophil Cytoplasmic Antibodies

Antineutrophil cytoplasmic autoantibodies (ANCA) are directed against proteins predominantly expressed in neutrophils, and can be subdivided based on indirect immunofluorescence into “cytoplasmic” (cANCA), “perinuclear” (pANCA), and “atypical” ANCA (24). The presence of ANCA has been reported in several diseases. In patients with (primary) systemic vasculitis, ANCA antibodies are usually directed against proteinase 3 (PR3) or myeloperoxidase (MPO) (25). However, some patients with systemic vasculitis (other than CF-associated forms) are negative for both anti-PR3 and anti-MPO, while they are positive for anti-bactericidal permeability increasing protein (BPI) ANCA, suggesting that the presence of the latter may be involved in the pathogenesis of this disease, too (26, 27). The bactericidal permeability increasing protein is an endotoxin-binding host protein present in azurophilic granules of neutrophils, which protects against Gram-negative bacteria infections. Some studies suggest that ANCA, and especially auto-antibodies directed against BPI, may play a role in increased bacterial colonization in CF (28–30). In fact, the lungs of pwCF can be colonized by a variety of bacteria, such as *Pseudomonas aeruginosa*, an opportunistic Gram-negative bacterium that does not normally cause respiratory disease in healthy individuals, but is the major respiratory pathogen in pwCF, partly because of impaired mucociliary clearance of thickened mucus secretions (29, 31). Thus, anti-BPI ANCA positivity in pwCF (or other conditions such as primary vasculitis) may contribute to bacterial colonization and/or susceptibility to infection.

**Figure 2:**
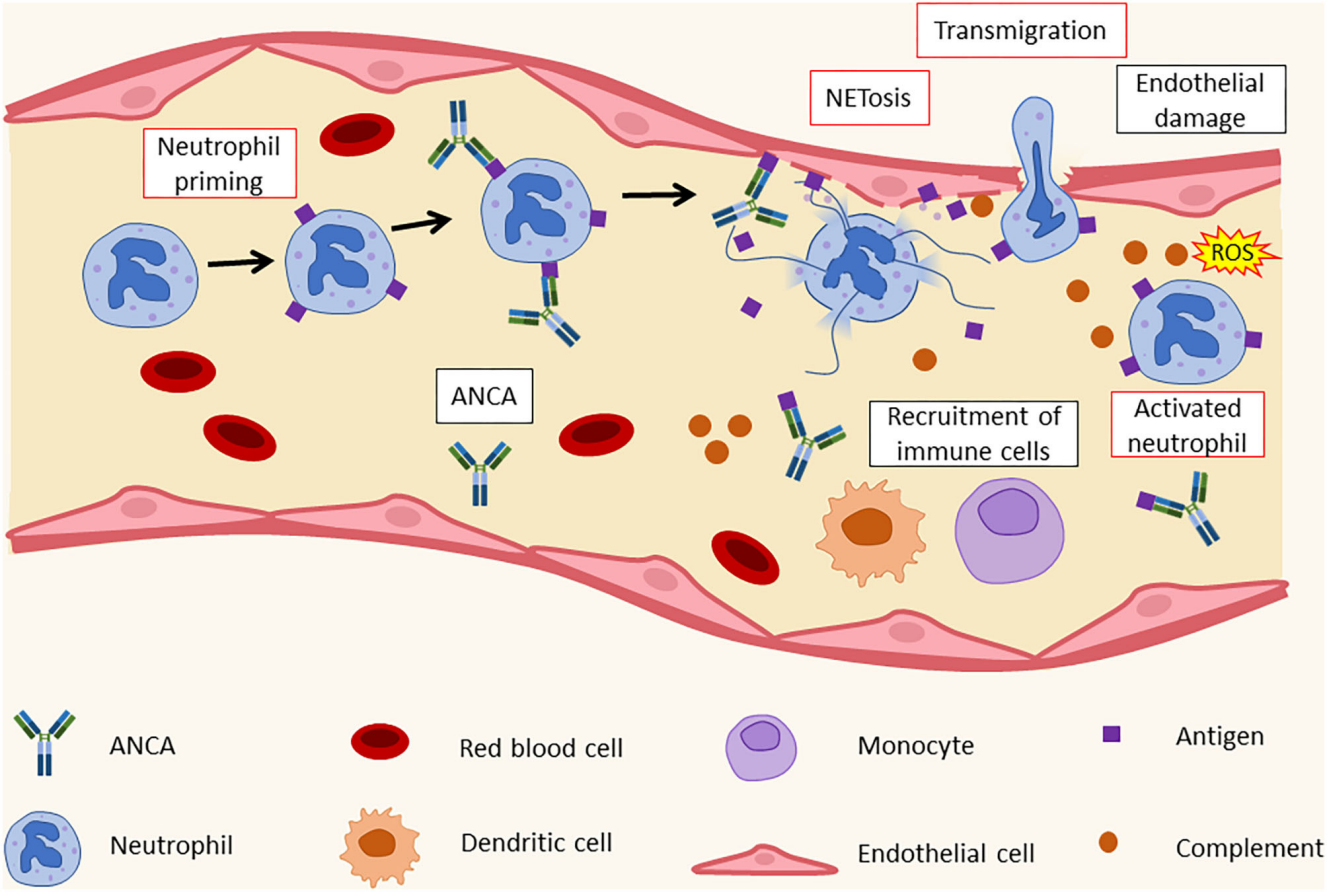
Histopathology of leukocytoclastic vasculitis. ANCA recognize their targets on activated neutrophils' surface. This leads to the formation of immunocomplexes and to an increase in adhesion molecule expression by neutrophils. As a result, neutrophils bind to endothelial cells and release NETs that cause endothelial damage and the recruitment of additional immune cells, such as dendritic cells (DCs), monocytes, and other neutrophils. ANCA, antineutrophil cytoplasmic antibodies; ROS, reactive oxygen species.

However, ANCA positivity may not only contribute to bacterial colonization/infection in CF and other conditions, but the resulting increased presence of pathogens may also prime for autoantibody generation. In pwCF, chronic bacterial respiratory infection and colonization results in continuous neutrophil recruitment into the lungs. The long-term presence of neutrophils, and the release of neutrophil extracellular traps (NETs, also see below) and other neutrophil components likely contribute to autoimmune/inflammatory responses (32). Subsequent generation of immune complexes (also see above) containing neutrophil components promotes tissue inflammation and damage (33) (Figure 2). Evidence supporting this comes from several observational studies. Neutrophil elastase (NE) activity in bronchoalveolar lavage fluid in the first 3 months of life, generally before respiratory symptoms are present, is a known risk factor associated with early bronchiectasis in children with CF (34). Similarly, children with CF frequently exhibit anti-BPI antibody positivity before developing clinical signs of CF, with children positive for autoantibodies developing worse respiratory disease than ANCA negative children (13). Furthermore, the presence of ANCA in children with CF is associated with indirect measures of disease severity, including low forced expiratory volume (FEV1), *P. aeruginosa* colonization, presence of multi-drug resistant bacteria or pan-resistant *P. aeruginosa*, the number of antibiotic courses received, the presence of CF-related liver disease, hypergammaglobulinemia, elevated C-reactive protein, and low BMI (28). ANCA directed against BPI, PR3, and/or MPO are also found in the serum of adult pwCF (28, 35).

**Figure 3:**
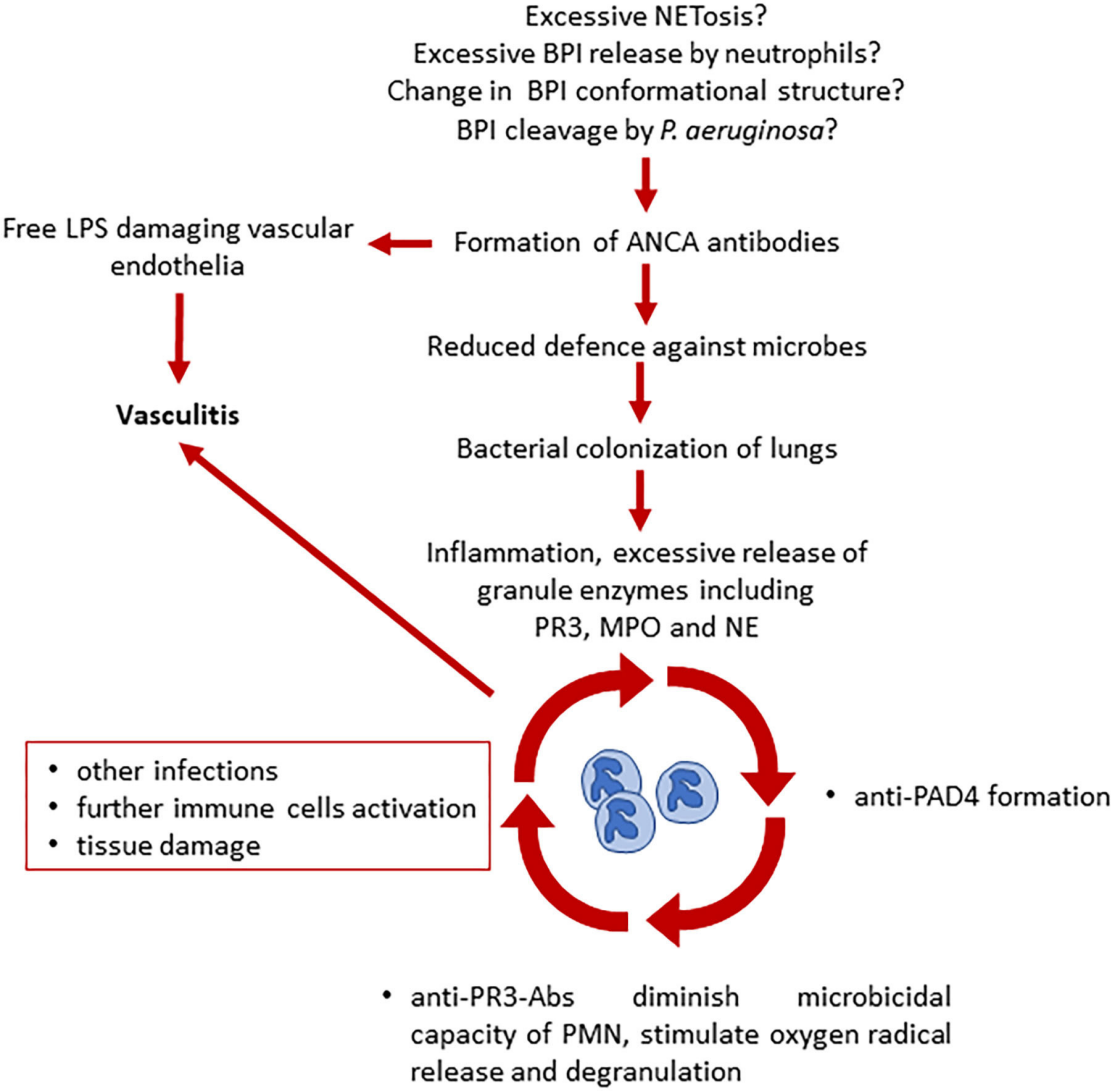
Proposed model of ANCA involvement in the development of vasculitis and chronic inflammation in CF (13, 33). Several hypotheses have been made on what triggers ANCA production in pwCF. ANCA presence leads to a reduced defense against microbes and to vascular endothelial damage due to free LPS. Bacterial colonization leads to further neutrophil activation, inflammation, and increased release of neutrophil granules. Here starts the vicious cycle that leads to additional ANCA formation, decrease of microbicidal capacity, immune cells activation, and tissue damage, causing vasculitis. BPI, bactericidal permeability increasing protein; LPS, lipopolysaccharide; ANCA, antineutrophil cytoplasmic antibodies; PAD4, peptidyl arginine deiminase 4; PR3, proteinase 3; MPO, myeloperoxidase; NE, neutrophil elastase.

The role of ANCA in CF, bacterial infections and vasculitis is complex. Currently, it is not entirely clear how ANCA are induced and whether *P. aeruginosa* airway colonization contributes to or is promoted by the presence of ANCA, including BPI-ANCA. Šedivá et al. suggested that the presence of ANCA may be a key, initial step for bacterial overgrowth. They suggest that CFTR channel mutations alter cell pH and charge in epithelial cells and neutrophils (that also express CFTR) (36, 37). As BPI is a cationic protein, neutrophils from pwCF may release more or structurally altered BPI when compared with cells from healthy individuals (13, 38). BPI protein may then, together with lipopolysaccharide (LPS), be internalized by macrophages by pinocytosis and presented to immune cells. These, in turn, may contribute to the generation of anti-BPI antibodies (13). This complex process could start the vicious cycle of inefficient immune response and subsequent colonization of airways with *Pseudomonas spp*. The same group suggested that induction of anti-PR3 ANCA antibodies happens later, after chronic inflammation and neutrophil accumulation/activation has been established as a result of excessive granule enzyme release (Figure 3) (13).

Another possible trigger for the production of ANCA antibodies in pwCF is the presence of *P. aeruginosa* which can cleave BPI in the CF airway. BPI cleavage may lead to the production of new epitopes that can stimulate anti-BPI antibody production resulting in auto-immune responses as well as reduced eradication of *Pseudomonas spp*. (29). Thus, bacterial colonization may represent an early event priming ANCA antibody generation. This is supported by the observation that CF patients who receive a sterile lung transplant, in the following, exhibit decreased levels of ANCA autoantibodies (39, 40).

Furthermore, aforementioned anti-BPI antibodies have the potential to block the protective effects of BPI against LPS which causes vascular endothelial cell injury and, in so doing, facilitates blood vessels inflammation and vasculitis (9).

Taken together, the presence of ANCA may contribute to the development of vasculitis in CF through allowing bacterial colonization/infection and associated damage, which contribute to IC generation and inflammation. As ANCA, at least to some extent, can also be the product of infection, inflammation and damage, the exact temporal and causative involvement of ANCAs in the inflammatory cascade causing CF-associated vasculitis is currently unknown.

### NETosis in CF Associated Vasculitis

Aforementioned anti-BPI antibodies activate neutrophils in the presence of TFN-α and induce NETosis (41). NETosis is a form of cell death that is distinct from others in that it causes the release of a lattice composed of DNA associated with citrullinated histones and granular cytoplasmic proteins. Physiologically, NETosis serves as an innate microbicidal mechanism.

**Figure 4:**
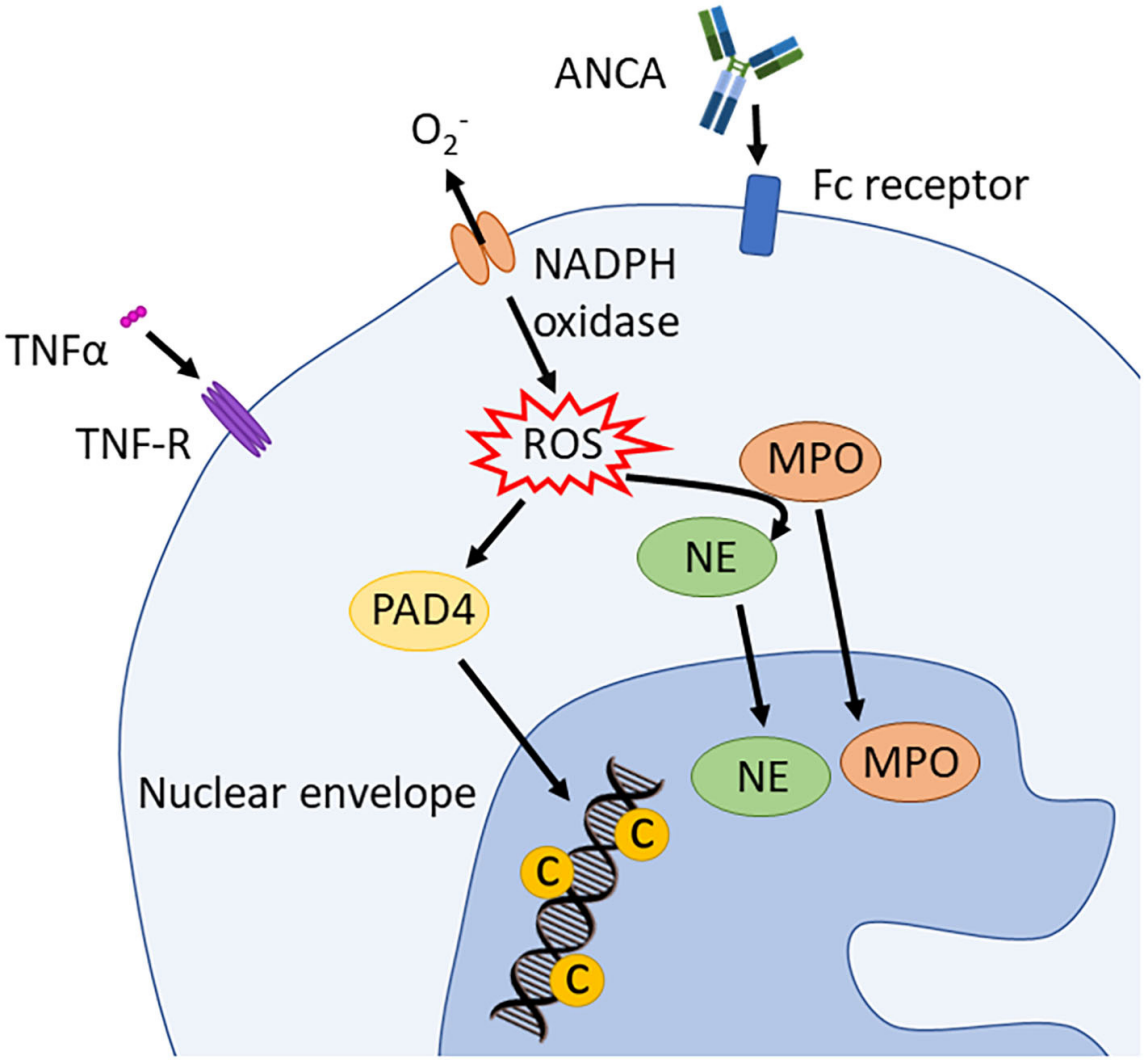
Induction of suicidal NETosis. NADPH oxidase assembles and activates the production of ROS. These stimulate PAD4 and, in an MPO dependent way, also NE activity. PAD4 *trans*-locates to the nucleus and hypercitrullinates histones that are simultaneously processed and cleaved by NE and MPO. This process leads to chromatin decondensation. ANCA, antineutrophil cytoplasmic antibodies; PAD4, peptidyl arginine deiminase 4; PR3, proteinase 3; MPO, myeloperoxidase; NE, neutrophil elastase; TNFα, tumor necrosis factor α; ROS, reactive oxygen spices; NADPH oxidase, nicotinamide adenine dinucleotide phosphate oxidase.

Suicidal NETosis, one of the three forms of NETosis (the others being vital NETosis and mitochondrial NETosis), depends on reactive oxygen species (ROS) and neutrophil elastase (NE) (42). During this process, the assembly of the neutrophil NADPH oxidase complex results in the production of ROS. Reactive oxygen species, as well as having intrinsic microbicidal activity, also stimulate the proteolytic activity of peptidyl arginine deaminase 4 (PAD4) and NE proteins in a myeloperoxidase (MPO)-dependent manner. Neutrophil elastase binds to and degrades F-actin, an essential component of eukaryotic cytoskeleton, in order to translocate, together with MPO, to the nucleus (42, 43). There, PAD4 hyper-citrullinates core histones that are simultaneously processed and cleaved by MPO and NE (Figure 4) (42, 43). This process leads to the decondensation and mobilization of chromatin through nucleosome rearrangement. Furthermore, during NETosis, NE is involved in nuclear envelope disintegration which allows the “mixing” of nucleic acids and granule proteins. NETs are thus composed of decondensed chromatin, and granular and cytoplasmic proteins such as NE, MPO, PR3, and BPI, and they are released from single perforations in the plasma membrane (42). Additionally, NE is important for the immobilization of neutrophils at the site of the infection, as it initiates actin cytoskeleton disassembly (43). Thrombocytes actively contribute to this process by expressing toll-like receptor (TLR)-4 through which they bind neutrophils and induce NETs formation (44).

Extracellular NETs, apart from having microbicidal activity, also play a role in limiting inflammation through cytokine and chemokine degradation (42). Thus, NETosis is a key component of physiologic immune homeostasis. However, in situations of uncontrolled NETosis, it can cause tissue damage, cardiovascular-thrombotic disorders, carcinogenesis and, indirectly, can contribute to many autoimmune and/or inflammatory diseases, including CF. NETs can prime macrophages and induce the release of TNF-α and IL-6, two potent pro-inflammatory cytokines. Particularly, it has been reported that neutrophils expressing defective CFTR show delayed apoptosis, that may well contribute to uncontrolled systemic inflammation and neutrophil dysfunction in CF (45–47). Impairment of physiologic cell death leads to a more efficient formation of NETs by neutrophils from pwCF when compared to healthy controls. This is further supported by the presence of extracellular human DNA in the airways of pwCF (48).

As mentioned above, the PAD4 enzyme citrullinates histone arginine residues. This is required for NET formation, and associated DNA decondensation and release by neutrophils. Recently, the presence of anti-PAD4 antibodies has been associated with CF (33). Antibody presence correlates with reduced lung function and the presence of *P. aeruginosa*. Their presence, however, is not correlated with genotype, age, sex, and bacteria mucoid status (33). Anti-PAD4 antibodies may have a relevant clinical effect in CF as they promote NETosis induced both by microbes and neutrophil malfunction (32). As the physiologically intracellular protein PAD4 is released from neutrophils during NETosis, it could act as a neoantigen and have a role in the development of autoimmune-like symptoms, just like the aforementioned BPI (33). Notably, anti-PAD4 antibodies are associated with poor joint prognosis in adult rheumatoid arthritis (RA) where they are present in up to 40% of patients (49). This may be of particular interest, as RA can be complicated by pulmonary involvement and vasculitis. In particular, RA-associated lung involvement associates with anti-PAD4 antibody positivity together with increased neutrophil activation (50, 51). However, to our knowledge, the exact involvement of anti-PAD4 antibodies in the pathogenesis of vasculitis has not been revealed yet.

Notably, NET formation can be seen at the site of ANCA-associated vasculitis lesions, together with the presence of myeloid dendritic cells (DCs). DCs are antigen presenting cells: they engulf, process and present antigens to T cells, thereby linking innate and adaptive immune responses. NETs activate DCs and trigger the autoimmune response against proteins released during NETosis, such as PR3, MPO, and BPI. ANCA, which are involved in the development of vasculitis, target neutrophil-derived proteins and further activate NETosis and the immune activation (42). Thus, the presence of ANCA in CF is, to some extent, both the cause and effect of NETosis.

One additional key factor contributing to aberrant NETosis in CF is the absence of a functional regulator of the TNF-α signaling pathway: CFTR (52). In fact, CFTR is involved in the regulation of TRADD (TNFR1-associated death domain protein) as it enhances its lysosomal degradation (52). In the absence of a functional CFTR, TRADD fails to be degraded and causes uncontrolled TNF-α signaling and NFκB activation which both mediate inflammation and facilitate NETosis (52).

While increased and poorly controlled NETosis is a hallmark of vasculitis in CF, it is also ineffective. The absence of a functional CFTR chloride channel impairs the microbicidal activity of the MPO product hypochlorous acid (HOCl), an important ROS involved in anti-bacterial responses (53). Secondly, in an inflamed environment, bacteria with a highly versatile genome such as *P. aeruginosa*, may become resistant to NETs, stimulate NETs production and use these to “hide” and evade the host's immune response (54).

Taken together, while still under investigation, increased NETosis in CF may be the result of reduced or absent CRTF function, bacterial colonization/infection, and/or the presence of ANCA autoantibodies. Increased but ineffective NETosis may contribute to vasculitis in pwCF through increased monocyte/macrophage activation and pro-inflammatory cytokine release, thrombocyte activation and the induction of pro-thrombotic cascades, insufficient control of bacterial colonization/infection, and the induction of tissue damage (and resulting inflammation, including IC generation).

### Inability to Suppress Neutrophil Activity in pwCF

Another factor promoting systemic inflammation and damage in CF is increased thrombocyte activation. CFTR is expressed by thrombocytes, where it is required for the release of mediators involved in inflammatory responses. In fact, reduced or absent CFTR function is associated with reduced Lipoxin A_4_ (LXA_4_) production (55). LXA_4_ is an anti-inflammatory mediator that is produced during platelet-monocyte interactions. It limits neutrophil activation, chemotaxis, adherence and *trans*-migration while it increases the uptake of apoptotic neutrophils by monocytes/macrophages (55). Additionally, LXA_4_ may limit the expression of pro-inflammatory IL-1β and TNF-α and of neutrophil chemotactic factor IL-8, while promoting the production of immune-regulatory IL-10 (55, 56).

**Figure 5:**
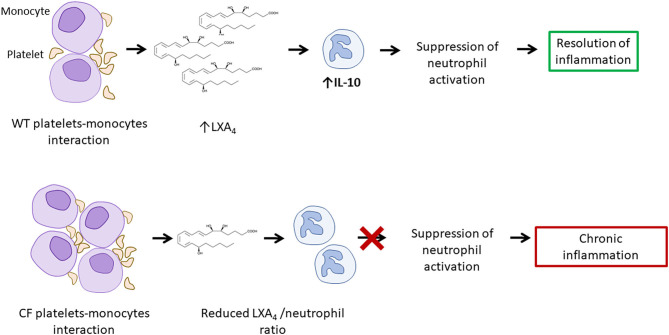
Increased platelet activation and reduced LXA_4_/neutrophil ratio result in increased neutrophil activity. Platelets-monocytes interaction leads to the production of LXA_4_, involved in the suppression of neutrophil activation. A reduced LXA_4_/neutrophil ratio in CF and may contribute to chronic inflammation and to the development of vasculitis. LXA_4_, lipoxin A_4_.

Although, in pwCF, thrombocytes aggregate more easily (potentially also as a result of aforementioned increased NETosis) and have increased capacity to form complexes with monocytes compared with healthy individuals, they produce reduced amounts of LXA_4_ and fail to contribute to the resolution of inflammation (57). This process may enhance the vicious cycle that causes chronic inflammation in CF and potentially contributes to the development of vasculitis (Figure 5).

### Inflammasome Activation in CF

In CF, epithelial and innate immune cells exhibit dysregulated signaling pathways which result in altered cell activation. Apart from regulating chloride transport, CFTR also influences additional ion channels' activity and thereby pro-inflammatory cytokines expression. While it is known that increased intracellular Cl^−^ concentrations activate pro-inflammatory cytokine secretion, more recently, altered intracellular K^+^ and Na^+^ concentrations have been linked with NLRP3 (NOD, leucine rich repeat and pyrin domain-containing protein 3) inflammasome activation (58, 59). The NLRP3 inflammasome is a multiprotein oligomer expressed by immune, epithelial and endothelial cells. When activated, the cytoplasmic NLRP3 inflammasome assembles and results in the activation or pro-inflammatory caspase-1, allowing maturation and secretion of pro-inflammatory cytokines IL-1β and IL-18, and mediates an inflammatory form of cell death, referred to as pyroptosis (60–63).

As inflammasome activation and pyroptosis are involved in several systemic and organ-specific autoimmune/inflammatory conditions, as well as autoimmune/inflammatory phenomena of infectious disease, these mechanisms may also be considered in CF-associated inflammation and damage (64). Indeed, monocytes and bronchial epithelial cells from pwCF are characterized by increased secretion of these effector interleukins. Recently, a link between Na^+^ influx through the epithelial sodium channel (ENaC) and NLRP3 inflammasome activation has been suggested (65). Authors discuss that intracellular accumulation of Cl^−^ may cause enhanced ENaC-mediated sodium influx and, as a result, enhanced K^+^ efflux, subsequently triggering inflammasome activation (65). Additionally, when CFTR function was enhanced in CF monocytes by CFTR modulators (discussed below), NLRP3 activation normalized (66).

**Figure 6:**
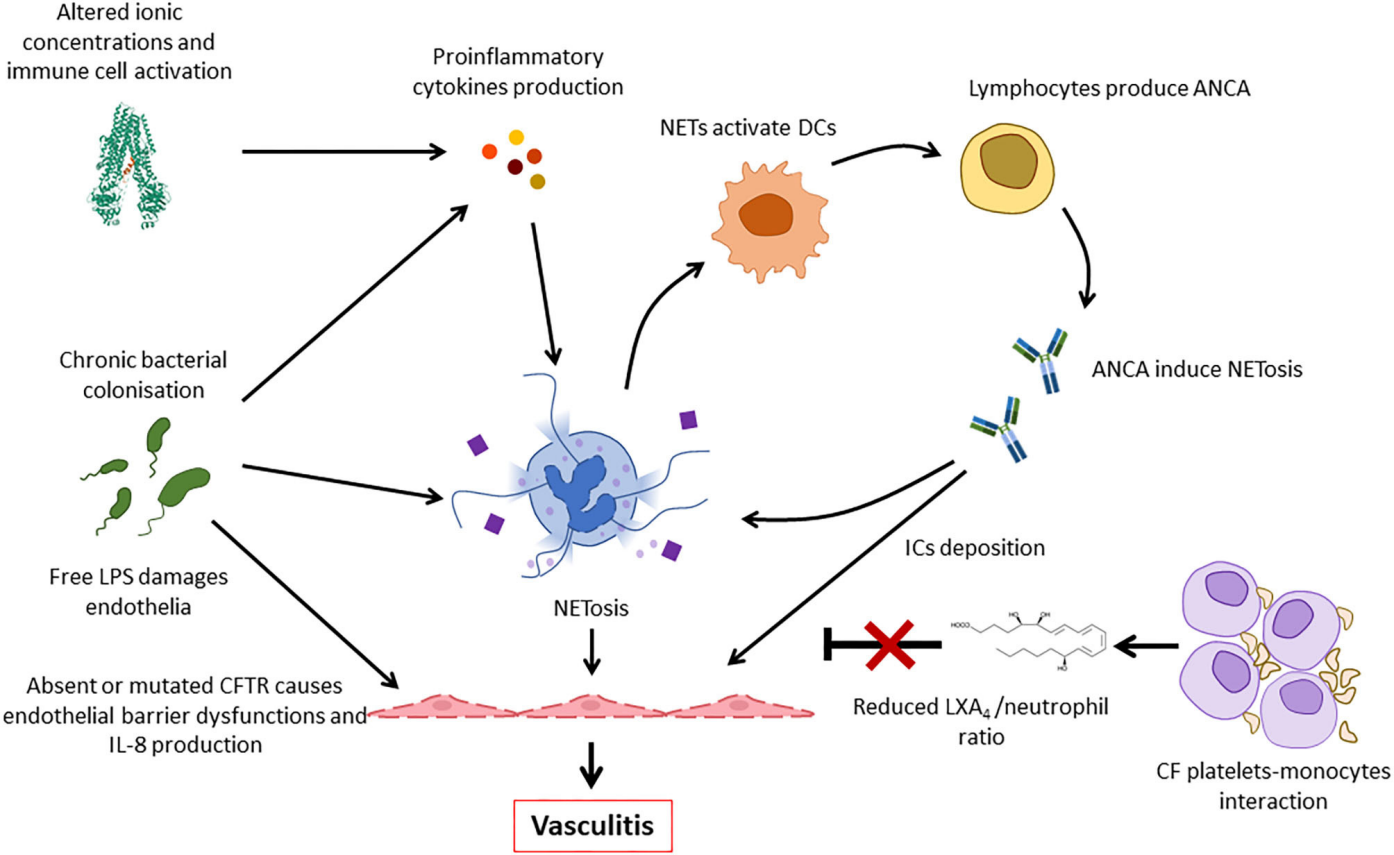
Pathogenic mechanisms involved in the development of vasculitis in CF. Deficiency or impaired function of CFTR leads to intracellular ionic alterations that can trigger inflammasome activation. This results in the release of pro-inflammatory cytokines that, together with persistent bacterial presence, induce NETosis. NETs activate dendritic cells (DCs) that stimulate ANCA autoantibody production that are involved in neutrophil activation and, possibly, immune complexes formation. Furthermore, thrombocytes form complexes with monocytes and fail to produce LXA_4_ that usually contributes to inflammation resolution. All these factors, and the increased release of IL-8 by endothelial cells contribute to continuous immune cell recruitment and NETosis, finally resulting in vasculitis. ANCA, antineutrophil cytoplasmic antibodies; LPS, lipopolysaccharide; DC, dendritic cell; NET, neutrophil extracellular trap; IC, immune complex.

As inflammasome activation and cytokine release contribute to tissue damage, neoantigen release and inflammation, aforementioned mechanisms, may contribute to vasculitis in CF (64, 67). Furthermore, endothelial NRLP3 inflammasome activation, which may be increased in CFTR deficient endothelial cells of pwCF, contributes to endothelial and coagulation system activation, and/or vasculitis (68). Endothelial activation induces the expression of cell adhesion molecules (CAMs), which are indeed increased in CF, and the increase of immune cell recruitment (69). Furthermore, the expression of a defective CFTR causes endothelial dysfunctions with impaired endothelial barrier function and stability after shear stress (70, 71). As a result, IL-8 release is enhanced by CF endothelial cells contributing to further neutrophil recruitment and activation (Figure 6) (71, 72).

Taken together, altered salt concentrations and disturbed ion flux in pwCF may result in increased inflammasome assembly, cytokine activation and release, inflammatory cell death (including autoantigen release), endothelial activation and immune cell migration, all of which may contribute to the inflammatory phenotype of CF and the development of CF-associated vasculitis.

## Clinical Considerations and Treatment Options

Although vasculitis occurs in only 2–3% of pwCF, case reports and case series, some of them admittedly published some years ago, report high associated mortality with 75–90% of pwCF dying within 2 years of vasculitis being diagnosed (8, 12). Most frequently, vasculitis affects the skin, but, in some cases it can also affect the brain, kidneys, and other organs (8). Published data is limited to case reports and small case series. Comparing 14 case reports published between 1979 and 2017 (Table 1), the majority of pwCF who developed vasculitis were adolescents or young adults (median: 22.5, range: 12–32) (8, 9). Based on reports available, vasculitis is associated with worsening of pulmonary symptoms and arthralgia. Though biopsy was not always performed, when it was, it showed typical features of leukocytoclastic vasculitis, including perivascular and sometimes interstitial inflammatory cell infiltrates, immunoglobulin and complement deposition in vessel walls, and cellular debris. A direct link between vasculitis and medical treatment was discussed only in two cases (8, 35), while Kayria et al. suggested a possible association between vasculitis and pulmonary colonization with *Burkordelia spp*. (74). Possible molecular similarities between microbial antigens and autoantigens, epigenetic variations that may lead to increased expression of autoantigens, immune complexes formation and deposition, and the stimulation of immune responses operated by NETs, have been discussed in this context (75, 76).

**Table 1:** Published case series and case reports from 1979 to 2017.

**References**	**Case**	**Sex**	**Age**	**ANCA**	**Co-infection**	**Arthralgia**	**Pre-existing medications**	**Diagnosis**	**Organ involvement**	**Treatment**	**Details and outcome**
Soter et al. (73)	1	M	16	nd	nd	+	nd	nd	Skin	Prednisone	No correlation with pulmonary exacerbation or drugs. Relapses. Death after two years
	2	M	20	nd	nd	+	nd	nd	Skin	None	Association with pulmonary exacerbation. No relapses. Death after 7 months
Fradin et al. (11)		F	18	nd	*P. aeruginosa*	+	Intravenous antibiotics	Leukocytoclastic vasculitis	Skin	Antibiotics	Association with pulmonary exacerbation. Deposits of C3 in blood vessel walls
Finnegan et al. (8)	1	M	32	–	*H. influenzae, S. aureus*	+	Pancreatic enzymes, carbamazepine, iron and folic acid supplements, salbutamol	Henoch-Schonlein purpura	Skin and kidneys	NSAIDs, then prednisolone + azathioprine, then prednisolone alone	Only one relapse, before steroid treatment
	2	M	19	–	*P. aeruginosa*	nd	Pancreatic enzymes, nebulized colomycin, vitamin supplements, salbutamol, colomycin	nd	Skin and brain	Prednisolone then switched to dexamethasone	No correlation with pulmonary exacerbation. Rash during final illness
	3	F	24	+	CMV, *P. aeruginosa*	nd	Pancreatic enzymes, vitamin supplements, salbutamol; tobramycin and carbenicillin then switched to colomycin	nd	Skin	Prednisolone	No relapses
	4	M	12	–	*P. aeruginosa*	nd	Pancreatic enzymes, vitamin supplements, ranitidine, glibenclamide, terbutaline, ferrous sulfate and magnesium supplements; gentamicin, ceftazidime and flucloxacillin switched to gentamicin, ticarcillin, flucloxacillin	Leukocytoclastic vasculitis	Skin	Ciprofloxacin and flucloxacillin	Correlation with pulmonary exacerbation. One relapse. The rash persisted for four months but cleared after the withdrawal of ranitidine
Wujanto and Ross (12)		M	22	nd	*S. aureus, P. aeruginosa*	nd	nd	nd	Skin	Azathioprine	Correlation with pulmonary exacerbation. 3 relapses before death
Molyneux et al. (35)		M	28	–	nd	+	Sulphasalazine, intrevenous antibiotics	Leukocytoclastic vasculitis	Skin	Chloroquine and prednisolone	Correlation with pulmonary exacerbation. One relapse
Ruiz Beguerie and Fernandez (9)		M	18	+	nd	nd	Tacrolimus and systemic corticosteroids at low doses, ciprofloxacin, azithromycin and colistin	Leukocytoclastic vasculitis	Skin	Rest and elevation of lower limbs	Correlation with pulmonary exacerbation. Was immunosuppressed with tacrolimus and systemic corticosteroids at low doses. One relapse. C3 deposits
Kayria et al. (74)	1	F	26	–	*B. cenocepacia*	nd	nd	Leukocytoclastic vasculitis	Skin	Dapsone, then switched to oral steroids, then to protopic 0.03% topically	No change with dapsone or oral steroids. Improvement with topical protopic but no complete resolution
	2	F	23	–	*B. cenocepacia, P. aeruginosa, S. aureus*	+	nd	nd	Skin	Oral steroids, then switched to azathioprine	No response to steroids. Rash responded to azathioprine and then resolved
	3	M	32	–	*B. cenocepacia*	nd	nd	Capillaritis	Skin	Compression stockings, NSAIDs and betnovate cream	Improvement with Non-steroidal anti-inflammatory medications and topical betnovate but no resolution
	4	F	26	–	*B. cenocepacia, P. aeruginosa*	+	nd	Erythema nodosum	Skin	NSAIDs	No association with infective exacerbations. Relapsing and remitting

*ANCA, antineutrophil cytoplasmic antibodies; F, female; M, male; +, presence; –, absence; nd, not determined*.

Detailed clinical and laboratory features have been reported for four patients who all exhibited hypergammaglobulinemia, potentially contributing to IC generation and deposition (8). One patient exhibited thrombocytosis (509.000/ml), supporting the aforementioned hypothesis that platelets play a role in the development and persistence of inflammation, and therefore the development of vasculitis in CF (8, 57).

The reported extent and clinical course of vasculitis in CF is variable. In some cases, vasculitis was limited to the skin, and some vasculitic rashes disappeared spontaneously within weeks. Physical rest and the use of compression stockings or symptomatic treatment was recommended in some cases, treatment with corticosteroids or NSAIDs was used in others (8, 9, 74). In other individual cases, vasculitis was associated with pulmonary deterioration, and skin lesions resolved with the improvement of lung disease following antibiotic therapy (8, 11). Unfortunately, relapses were common and required immune modulating treatment in some patients (8, 12). Used agents included prednisolone, alone or in combination with other immunomodulators, such as azathioprine and other non-biologic/conventional disease-modifying anti-rheumatic drugs (namely methotrexate and mycophenolate mofetil), and/or antibiotics (8, 12). While aforementioned drugs may be used as maintenance treatment in CF-associated vasculitis, reliable evidence (e.g., from randomized studies) supporting their efficacy and safety currently does not exist.

Based on experience with other forms of vasculitis, in patients with steroid resistant cutaneous vasculitis, the antimalaria agents (chloroquine and hydroxychloroquine) may be used to maintain remission and reduce flare frequency (35). Several characteristics make antimalarial agents a potential treatment for vasculitis: they reduce circulating cytokines levels (e.g., IL-1 and IL-6) and the production of TNF-α in PBMCs, and stabilize endothelial membranes which results in the improvement of endothelial function (77).

The use of immune modulating treatments in CF is not without risk. While they are established and efficacious medications and frequently used in systemic autoimmune/inflammatory diseases, experience in CF is limited. As they dampen immune responses and inflammation, they may increase the risk of infections or malignancy, both a pre-existing concern in pwCF (78). Possible alternative therapies may involve treatments targeting individual cytokines, such as IL-1 inhibitors. Indeed, preliminary evidence from mouse models suggests beneficial effects of IL-1-directed treatments limiting inflammation in CF (79).

Recently, a new class of medications has become available for the treatment of CF (80, 81). So-called CFTR modulators enhance CFTR activity and increase its presence within the cell membrane. It has been demonstrated that CFTR modulator treatment has anti-inflammatory effects (66). Monocytes from pwCF carrying the most frequent *CFTR* p.Phe508del (ΔF508 *CFTR*) mutation show an increased production and activation of IL-1β and IL-18 through inflammasome assembly and pro-inflammatory caspase-1 activation, as well as TNF-α expression when compared with monocytes from healthy controls. Treatment of patient monocytes with combinations of CFTR modulators, ivacaftor/lumacaftor and ivacaftor/tezacaftor, leads to a reduction of caspase-1 activation, subsequently reduced IL-18 and TNF-α secretion, and increased immune-regulatory IL-10 production (66). Based on these observations, CFTR modulators may, in addition to resolving underlying molecular pathological mechanisms in CF, also inhibit inflammasome-mediated inflammation, and thus reduce the incidence of vasculitis in pwCF. However, effectiveness and safety of these medications in CF-related vasculitis requires further investigation, particularly because of the potential for drug interactions.

## Conclusions

Systemic inflammation is a hallmark of CF. Vasculitis is a rare, but potentially severe complication of CF as it associates with poor prognosis. Where available, biopsies show evidence of leukocytoclastic (immune complex) vasculitis. Several hypotheses have been developed regarding possible underlying molecular causes, which include immune complex formation in the presence of pathogens, therapeutics, and/or autoantibodies (namely antineutrophil cytoplasmic antibodies; ANCA). ANCA autoantibodies may limit the immune system's ability to successfully clear pathogens and to terminate inflammatory responses. However, ANCA may also be the product of uncontrolled inflammation, potentially inducing a vicious circle. Another mechanism that likely contributes to vascular inflammation is NLRP3 inflammasome activation in innate immune cells and endothelia, both of which may contribute to endothelial activation, tissue damage, and immune complex formation and deposition.

Clinical reports and published evidence on therapeutic approaches are sparse. Most patients are treated with antibiotics to clear pathogens, and NSAIDs, corticosteroids, and/or chloroquine to control inflammation. If on withdrawal of corticosteroids symptoms recur, classical DMARDs, such as azathioprine may be considered. However, immunosuppression may increase the risk of infection and/or malignancy. “New” therapeutic options may include cytokine-blocking agents (e.g., IL-1 blockade). Recently licensed and future CFTR modulators may transform treatment and prognosis in CF but a deeper understanding of the underlying causes of inflammation and individual factors associated with different phenotypes is crucial to develop target-directed and individualized treatments for CF and associated immune diseases.

Further studies, involving both pediatric and adult CF patients, are urgently needed to identify causative connections between bacterial colonization and/or infection, tissue damage, the presence of autoantibodies and immune complexes, altered NETosis, inflammasome activation, and (potentially) additional mechanisms that contribute to disease pathology and phenotype. This will allow future individualized and target-directed approaches to treat CF patients effectively and with reduced treatment-associated side-effects.

## Author Contributions

All authors listed have made a substantial, direct, and intellectual contribution to the writing of the manuscript interpreting the relevant literature, drafting the article, and revising it critically.

## Conflict of Interest

The authors declare that the research was conducted in the absence of any commercial or financial relationships that could be construed as a potential conflict of interest.
